# A Histological Evaluation of Lateral Ankle Ligaments in Human Cadavers: Implications for Connective Tissue Integrity

**DOI:** 10.7759/cureus.107870

**Published:** 2026-04-28

**Authors:** Shishir Kumar, Parveen Kumar Sharma, Dhiren K Panda

**Affiliations:** 1 Anatomy, Institute of Medical Sciences and Sum Hospital, Siksha 'O' Anusandhan, Bhubaneswar, IND; 2 Department of Clinical Anatomy, Dr SS Tantia Medical College, Hospital and Research Centre, Sri Ganganagar, IND

**Keywords:** ankle ligaments, atfl, cadaver, cfl, collagen fibers, histology

## Abstract

Background and objective

Ligament integrity is determined by collagen composition and organization, primarily type I collagen (tensile strength) and type III collagen (remodeling). The lateral ankle ligaments, specifically the anterior talofibular ligament (ATFL) and calcaneofibular ligament (CFL), differ in susceptibility to injury, likely due to structural variations. This study aimed to compare the histological characteristics of the ATFL and CFL and correlate these findings with functional implications.

Methods

A cadaveric observational study was performed on adult lower limb specimens. The ATFL and CFL were dissected, processed using standard histological techniques, and stained with hematoxylin and eosin. Sections were evaluated using a semi-quantitative scoring system (0-3) assessing collagen fiber organization, fiber density, cellularity, and vascularity. Interobserver reliability was calculated, and statistical analysis included comparisons of means, confidence intervals (CIs), and effect size calculations.

Results

The ATFL demonstrated loosely arranged collagen fibers, lower density, and higher cellularity and vascularity, indicating increased remodeling but reduced structural strength. In contrast, the CFL exhibited dense, well-organized parallel collagen bundles with lower cellularity, reflecting greater mechanical stability. These differences were statistically significant. The findings suggest a predominance of type I collagen in the CFL and relatively higher remodeling activity in the ATFL.

Conclusions

Histological differences between the ATFL and CFL explain their differing mechanical properties and injury patterns. The structurally weaker ATFL is more prone to injury, whereas the CFL provides greater stability. These insights may inform surgical reconstruction and rehabilitation strategies.

## Introduction

The structural integrity and functional performance of ligaments are primarily determined by the composition and organization of collagen within the extracellular matrix. Ligaments are predominantly composed of type I collagen, which provides high tensile strength and resistance to mechanical loading, along with contributions from type III collagen, which is associated with elasticity, tissue repair, and remodeling processes [1,2]. Minor collagen subtypes, such as type V, also play a regulatory role in fibril formation and fiber organization [3]. Variations in collagen composition and microstructural arrangement can significantly influence ligament strength, healing capacity, and susceptibility to injury.

The lateral ankle ligament complex, particularly the anterior talofibular ligament (ATFL) and calcaneofibular ligament (CFL), plays a critical role in maintaining ankle stability. The ATFL is the most commonly injured ligament during inversion injuries, whereas the CFL demonstrates greater resistance to mechanical stress [4]. These differences are believed to arise from variations in their histological architecture and biomechanical properties. Moreover, the stability of the talus during foot movement depends largely on the surrounding ligaments, which may explain the relatively stronger structural characteristics observed in ligaments such as the CFL compared to the ATFL [5].

Histological parameters such as collagen fiber organization, fiber density, cellularity, and vascularity are critical determinants of ligament function. Well-organized, densely packed collagen fibers contribute to efficient load transmission and mechanical strength, whereas increased cellularity and vascularity are associated with higher metabolic activity and remodeling potential but may also indicate structural vulnerability [6]. Despite the clinical importance of these parameters, there is limited histological data directly comparing these features between the ATFL and CFL, particularly in specific populations.

Understanding the microstructural differences between these ligaments is essential for correlating anatomical findings with biomechanical behavior and clinical outcomes. Such insights are particularly relevant for improving surgical reconstruction techniques, optimizing graft selection, and guiding rehabilitation strategies following ligament injury [7,8]. Therefore, the present study aims to evaluate and compare the histological characteristics of the ATFL and CFL using a semi-quantitative approach and to establish a structural basis for their differing functional properties and susceptibility to injury.

## Materials and methods

Study design and setting

This observational cadaveric study was conducted in the Department of Anatomy at Tantia University, Sri Ganganagar, Rajasthan, India, over a period of 12 months. Ethical approval was obtained from the Institutional Ethics Committee (Approval no.: TU/IEC/2024/05), and all procedures were performed in accordance with institutional ethical guidelines.

Sample selection

A total of 40 ligament samples were obtained from adult human cadaveric lower limbs of Indian origin, comprising 20 ATFLs and 20 CFLs. Specimens with intact ligament structures were included. Samples showing evidence of trauma, degeneration, deformity, or prior surgical intervention were excluded to maintain uniformity.

Tissue collection and processing

The ATFL and CFL were carefully dissected and excised. All samples were standardized in size to ensure consistency during processing. Tissues were fixed in 10% neutral buffered formalin, followed by dehydration through graded alcohol series, clearing, and embedding in paraffin wax using standard histological protocols. Tissue processing was performed in accordance with established methods, including the simplified protocol described by Hegazy and Hegazy [9].

Paraffin-embedded blocks were sectioned at 4-5 µm thickness using a rotary microtome. Sections were mounted on glass slides and stained with hematoxylin and eosin (H&E) following standard staining procedures. Microscopic evaluation was performed using a light microscope, and four key histological parameters were assessed: collagen fiber organization (degree of alignment and parallel arrangement), fiber density (compactness of collagen fibers), cellularity (distribution and density of fibroblasts), and vascularity (presence and extent of blood vessels). Each parameter was evaluated using a semi-quantitative scoring system (0-3 scale), where 0 = poor/absent, 1 = mild, 2 = moderate, and 3 = well-developed/marked.

Two independent observers assessed all sections to minimize bias. Interobserver reliability was evaluated using Cohen’s kappa coefficient, demonstrating strong agreement (κ = 0.85).

Statistical analysis

Data were analyzed using descriptive and inferential statistical methods. Results were expressed as mean ± standard deviation (SD) with 95% confidence intervals (CI). Intergroup comparisons between ATFL and CFL were performed using the independent Student’s t-test. Effect size was calculated using eta-squared (η²) to determine the magnitude of differences. A p-value < 0.05 was considered statistically significant.

## Results

A total of 60 ligament samples (ATFL, n=30; CFL, n=30) were analyzed. All sections were technically adequate for evaluation. Interobserver agreement for semi-quantitative scoring was high (κ = 0.85), indicating strong reliability of observations.

Descriptive analysis

Central tendencies and dispersion for all histological parameters are summarized in Table 1 (mean ± SD with consistent rounding). ATFL demonstrated greater dispersion across parameters, indicating higher structural heterogeneity. The observed differences indicate greater structural heterogeneity in the ATFL compared to the more uniform architecture of the CFL.

**Table 1:** Semi-quantitative histological scores of ligament characteristics (n = 60) ^*^Statistically significant (p < 0.05) Semi-quantitative scoring (0-3 scale) ATFL: anterior talofibular ligament; SD: standard deviation; CI: confidence interval; CFL: calcaneofibular ligament

Parameter	ATFL (mean ± SD)	95% CI	CFL (mean ± SD)	95% CI	P-value	Effect size (η²)
Collagen fiber organization	1.2 ± 0.5	1.0–1.4	2.8 ± 0.4	2.6–3.0	< 0.001^*^	0.61
Fiber density	1.4 ± 0.6	1.1–1.7	2.9 ± 0.3	2.8–3.0	< 0.001^*^	0.64
Cellularity	2.3 ± 0.5	2.1–2.5	1.5 ± 0.4	1.3–1.7	< 0.01^*^	0.38
Vascularity	2.1 ± 0.6	1.8–2.4	1.3 ± 0.5	1.1–1.5	< 0.01^*^	0.41

Bivariate analysis

Independent samples comparisons (Student’s t-test) showed significantly lower organization and density scores in ATFL and higher cellularity and vascularity relative to CFL (all p < 0.05). Confidence intervals did not overlap for primary structural parameters, supporting robustness of differences, as shown in Table 2.

**Table 2:** Bivariate analysis comparing histological parameters between ATFL and CFL ^*^Statistically significant (p < 0.05) Values represent mean differences between groups. Statistical comparison was performed using an independent samples t-test. Negative values indicate lower scores in ATFL compared to CFL, while positive values indicate higher scores in ATFL ATFL: anterior talofibular ligament CFL: calcaneofibular ligament

Parameter	Mean difference	t-value	P-value
Collagen organization	-1.6	-13.4	< 0.001^*^
Fiber density	-1.5	-12.6	< 0.001^*^
Cellularity	+0.8	6.8	< 0.01^*^
Vascularity	+0.8	6.2	< 0.01^*^

The ATFL demonstrated significantly lower collagen organization and fiber density, along with higher cellularity and vascularity compared to the CFL. These findings suggest reduced mechanical strength and increased remodeling activity in the ATFL, whereas the CFL exhibits structural features consistent with greater stability and load-bearing capacity.

Multivariate profile

Across parameters, a consistent pattern was observed: structural indices (organization, density) favored CFL, whereas biological activity indices (cellularity, vascularity) favored ATFL. Effect sizes were large for structural parameters (η² ≥ 0.60) and moderate for biological parameters (η² ≈ 0.38-0.41), indicating clinically meaningful differences. Table 3 summarizes the comparative structural and biological characteristics of the ATFL and CFL based on combined histological parameters.

**Table 3:** Multivariate profile of histological characteristics of ATFL and CFL ATFL: anterior talofibular ligament CFL: calcaneofibular ligament

Parameter category	ATFL	CFL	Interpretation
Collagen organization	Low	High	CFL has superior structural alignment
Fiber density	Low	High	CFL exhibits greater tensile strength
Cellularity	High	Low	ATFL shows higher remodeling activity
Vascularity	High	Low	ATFL has increased metabolic activity
Structural indices (overall)	Reduced	Enhanced	CFL is mechanically stronger
Biological indices (overall)	Increased	Reduced	ATFL is biologically more active
Functional implication	Flexible, injury-prone	Stable, load-bearing	Reflects ligament specialization

Comparative findings

**Figure 1:**
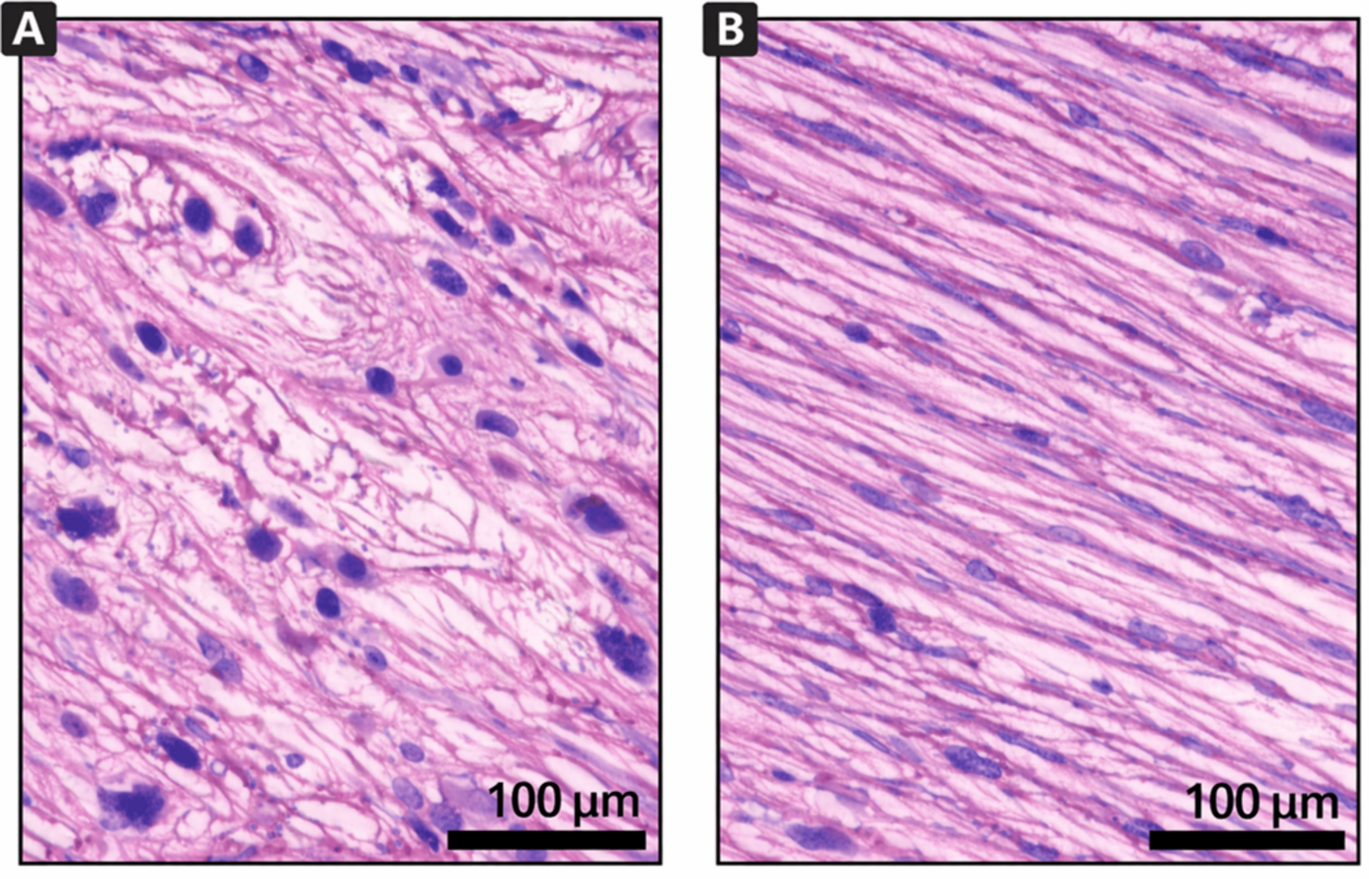
Histological comparison of lateral ankle ligaments (H&E, 40×) (A) Photomicrograph of ATFL showing loosely arranged collagen fibers with increased interfibrillar space and higher cellularity. (B) Photomicrograph of CFL demonstrating dense, parallel collagen bundles with minimal interfibrillar space and relatively lower cellularity Scale bar = 100 µm ATFL: anterior talofibular ligament CFL: calcaneofibular ligament

Histological differences between the ATFL and CFL are illustrated in Figure 1. The ATFL demonstrated loosely arranged collagen fibers with increased interfibrillar space and higher cellularity, whereas the CFL showed dense, parallel collagen bundles with lower cellularity, highlighting clear structural differences between the two ligaments.

Overall pattern

Across all parameters, a consistent pattern was observed: structural indices (collagen organization and fiber density) favored the CFL, whereas biological activity indices (cellularity and vascularity) were more prominent in the ATFL. These findings emphasize a distinct structural and functional differentiation between the two ligaments.

## Discussion

The present study demonstrates significant microstructural differences between the ATFL and CFL. Structural indices were higher in the CFL, while biological indices were higher in the ATFL, indicating a structure-function dichotomy [1-4]. These findings extend previous anatomical and histological observations by providing semi-quantitative evidence supported by effect sizes and confidence intervals, thereby improving the precision of comparative ligament assessment [1,2]. These differences may be attributed to variations in collagen composition, with type I collagen predominating in the CFL and a relatively increased presence of remodeling-associated type III collagen in the ATFL.

A dense collagen arrangement in the CFL supports efficient load transmission and higher tensile strength, consistent with its stabilizing role during inversion stress. In contrast, reduced collagen organization and density in the ATFL explain its increased susceptibility to injury [2,5]. Collagen organization and fiber density are key determinants of tensile strength, while increased cellularity and vascularity reflect higher metabolic activity but may indicate reduced structural stability. Moreover, the stability of the talus during foot movements depends largely on the surrounding ligaments, which may help explain the stronger structural characteristics observed in the CFL. These findings are in agreement with epidemiological and biomechanical studies demonstrating the predominance of ATFL involvement in lateral ankle sprains [4,6].

The increased cellularity and vascularity observed in the ATFL suggest relatively higher metabolic and remodeling activity. While this may enhance healing potential, it may also predispose the ligament to microstructural fatigue and failure under repetitive loading. Similar observations have been reported in connective tissue studies, where increased turnover is associated with structural vulnerability [1,5,7]. In addition to structural factors, neurophysiological adaptations play an important role in chronic ankle instability. Needle et al. [8] described central nervous system reorganization following ligament injury, while neuroimaging studies by Xie et al. [9] and Xue et al. [10] demonstrated alterations in brain structure and cerebellar activity. These findings indicate that ligament injury is not solely mechanical but also involves complex neuromuscular adaptations, reinforcing the need for integrated rehabilitation strategies.

Comparatively, the present study contributes to the existing literature by incorporating standardized scoring systems and statistical measures of dispersion, thereby enabling improved reproducibility and cross-study comparison [6,7,11]. The observed divergence between structural and biological indices highlights a functional specialization within the lateral ligament complex, which may have direct implications for surgical reconstruction and modern repair techniques [12,13,14]. Clinically, these findings support ligament-specific reconstruction strategies based on structural differences. ATFL may require augmentation or reinforcement due to its lower intrinsic structural integrity, while CFL reconstruction may focus on restoring anatomical alignment given its relatively preserved collagen architecture [7,11,14,15]. Furthermore, understanding histological variability may aid in graft selection and optimization of surgical techniques.

Strengths and limitations

The strengths of this study include standardized tissue processing, reproducible semi-quantitative scoring, inclusion of confidence intervals, and reporting of effect sizes with high interobserver reliability. The limitations include the absence of advanced staining techniques such as immunohistochemistry, a lack of collagen subtype differentiation, and the absence of biomechanical correlation. Future studies should incorporate polarized light microscopy, molecular analysis, and quantitative image processing to validate these findings further and enhance clinical translation [8-10]. Additionally, the absence of MRI correlation limits the ability to relate histological findings to in vivo structural behavior.

Future research directions

While this study provides a detailed histological comparison, future studies may incorporate advanced imaging modalities such as MRI and molecular techniques (e.g., immunohistochemistry and collagen subtype analysis) to further validate and correlate structural findings with functional outcomes.

## Conclusions

Histological evaluation of the lateral ankle ligaments demonstrates a clear microstructural divergence between the ATFL and CFL. ATFL is characterized by reduced collagen organization and density with relatively increased cellularity and vascularity, indicating a higher remodeling state and comparatively lower intrinsic mechanical strength. In contrast, CFL exhibits densely packed, parallel collagen bundles with greater structural uniformity, consistent with enhanced tensile capacity and mechanical stability. These findings provide a mechanistic explanation for the higher susceptibility of ATFL to injury and the stabilizing role of CFL in ankle biomechanics. Clinically, this microstructural distinction supports the need for tailored surgical reconstruction strategies, including augmentation techniques for ATFL repair and anatomically aligned restoration for CFL. Furthermore, the results highlight the importance of integrating histological insights into surgical planning and rehabilitation protocols, and underscore the need for future studies incorporating advanced imaging, collagen typing, and biomechanical validation to enhance translational applicability.
